# Nomogram for predicting the risk of postoperative delirium in elderly patients undergoing orthopedic surgery

**DOI:** 10.1186/s13741-024-00393-9

**Published:** 2024-05-04

**Authors:** Yunping Fan, Tingjun Yang, Yuhan Liu, Haibin Gan, Xiaohua Li, Yanrong Luo, Xuping Yang, Qianyun Pang

**Affiliations:** 1 Department of Anesthesiology, Shizhu Tujia Autonomous County People’s Hospital, Chongqing, 409100 China; 2 Department of Anesthesiology, Chongqing University Cancer Hospital, Hanyu Road No. 181, Shapingba District, Chongqing, 400030 China

**Keywords:** Orthopedic surgery, Postoperative delirium, Elderly patients, Predictive models

## Abstract

**Objective:**

To retrospectively analyze the risk factors for postoperative delirium (POD) after orthopedic surgery in elderly patients and establish an individualized nomogram to predict the risk of POD.

**Methods:**

The data of 1011 patients who underwent orthopedic surgery from January 2019 to January 2022 were retrospectively analyzed. Univariate and multivariate logistic analyses were used to screen for independent risk factors. Stepwise regression was conducted to screen risk factors to construct a nomogram to predict the risk of POD after orthopedic surgery in elderly individuals, and nomogram validation analyses were performed.

**Results:**

The logistic regression results showed that age (≥ 75 years old vs. < 75 years old; odds ratio (OR) = 2.889; 95% confidence interval (CI), 1.149, 7.264), sex (male vs. female, OR = 2.368; 95% CI, 1.066, 5.261), and preoperative cognitive impairment (yes vs. no, OR = 13.587; 95% CI, 4.360, 42.338) were independent risk factors for POD in elderly patients who underwent orthopedic surgery (*P* < 0.05). A nomogram was constructed using 7 risk factors, i.e., age, American Society of Anesthesiologists (ASA) classification, sex, preoperative hemoglobin (Hb), preoperative pulmonary disease, cognitive impairment, and intraoperative infusion volume. The area under the curve (AUC) showed good discrimination (0.867), the slope of the calibration curve was 1.0, and the optimal net benefit of the nomogram from the decision curve analysis (DCA) was 0.01–0.58.

**Conclusion:**

This study used 7 risk factors to construct a nomogram to predict the risk of POD after major orthopedic surgery in elderly individuals, and the nomogram had good discrimination ability, accuracy, and clinical practicability.

**Supplementary Information:**

The online version contains supplementary material available at 10.1186/s13741-024-00393-9.

## Introduction

The global population is aging, and the number of elderly patients requiring surgical treatment is increasing every year. Elderly patients are prone to undergo major orthopedic surgeries, such as hip and knee replacements and spine and fracture surgeries, and are also prone to postoperative delirium (POD) due to joint degeneration, underlying diseases, and frailty (Urban et al. 2020). POD is a common central nervous system complication in elderly patients, with an incidence of approximately 17.6% after major orthopedic surgery, and is often underdiagnosed (Rong et al. 2021). POD significantly affects patients’ postoperative recovery, prolongs the length of stay (LOS), increases medical costs and rehospitalization rates, and increases mortality rates (Lee et al. 2013; Susano et al. 2019).

POD is related to patient age, preoperative anxiety and cognitive impairment, education level, American Society of Anesthesiologists (ASA) classification, preoperative combined cardiopulmonary and brain diseases, anemia and blood transfusion, and preoperative nutritional status and is also related to the surgical approach, duration of surgery, anesthetic medication, analgesia, and postoperative pain (Urban et al. 2020; Susano et al. 2019; Chu et al. 2016; Liang et al. 2015; Yang et al. 2017; Ali et al. 2021). POD is the result of many factors; therefore, it is very important to accurately identify various perioperative risk factors, carry out risk stratification, and intervene.

Nomograms can be used to identify risk factors and perform risk stratification and are simple and effective. Predictive models for POD in elderly orthopedic surgical patients have been constructed in many studies. Kim EM et al. reported that preoperative delirium was the strongest predictor; however, only patients who underwent hip fracture surgery were included, and anesthesia factors were not included (Kim et al. 2020). Chen et al. constructed a predictive model for POD in elderly patients undergoing hip and knee replacement surgery, but the sample size was small, and the included indicators, such as cystatin C, are not commonly used in clinical practice (Chen et al. 2021). Zhang et al. constructed a prediction model of POD for elderly patients undergoing hip surgery; the model had a relatively low area under the curve (AUC) and an insufficient degree of fit (Zhang et al. 2019). Liang et al. constructed 2 models for predicting POD in elderly patients undergoing orthopedic surgery, but the sample size was small, and some risk factors, such as postoperative pain, were not included (Liang et al. 2015). To date, there is no good simple and effective model for predicting POD in elderly patients undergoing orthopedic surgery.

Therefore, this study retrospectively analyzed the general characteristics and surgical and anesthesia factors of elderly patients who underwent orthopedic surgery at our hospital to identify and assign risk factors for POD and constructed a nomogram of POD after orthopedic surgery in elderly patients, thus providing a reliable basis for assisting in the early clinical identification of high-risk patients and early preventive measures.

## Materials and methods

### Research design

This study was approved by the hospital’s medical ethics committee (Scientific Ethics Review No. 18 in 2022). The perioperative data of patients who underwent orthopedic surgery at Shizhu Tujia Autonomous County People’s Hospital, from January 2019 to January 2022, were retrospectively analyzed.

This was a retrospective study; when the data were collected, they were anonymized, i.e., names, hospital ID, and the date of operation were excluded. Therefore, informed consent was not necessary.

### Inclusion and exclusion criteria

The inclusion criteria were as follows: (1) age ≥ 65 years and (2) major orthopedic surgery including hip replacement, knee replacement, and spine and limb fracture surgery. The exclusion criteria were as follows: (1) severe combined injury; (2) superficial minor operations, such as debridement and suturing of superficial wounds and mass excision; and (3) incomplete clinical medical records.

### Data extraction

Data were extracted from patients’ electronic medical records, anesthesia records, test records, inspection reports, and nursing records.

Observation indicators included (1) patient conditions [age, sex, ASA classification, New York Heart Association (NYHA) classification, education level, hearing level, history of smoking, history of alcoholism, cardiovascular complications (coronary heart disease, hypertension, arrhythmia, etc.), diabetes, preoperative pulmonary diseases (chronic obstructive pulmonary disease (COPD), asthma, silicosis and pulmonary infection), preoperative cognitive impairment, and preoperative biochemical tests, including hemoglobin (Hb), white blood cells (WBC), creatinine (Cr), and neutrophil-to-lymphocyte ratio (NLR)] and (2) surgical and anesthesia factors [surgical site, operation time, anesthesia method, blood loss, fluid volume, blood transfusion, transfer to intensive care unit (ICU) after surgery, postoperative analgesia, and pain score within 48 h after surgery].

### Outcomes

The primary outcome was POD, and the secondary outcome was the postoperative length of hospital stay (LOS).

POD is diagnosed using “The Diagnostic and Statistical Manual of Mental Disorders, Fifth Edition” (Diagnostic and statistical manual of mental disorders fifth edition [M].Arlington VS:American Psychiatric Association 2013). The diagnosis includes (1) attention and consciousness disorder; (2) short disorder duration (usually hours to days), with fluctuating severity over the course of 1 day; (3) additional cognitive impairment (such as memory deficit, disorientation, visual or language impairment); (4) attention deficit, disorder of consciousness, and cognitive dysfunction that cannot be explained by the primary disease or known cognitive impairment; and (5) medical history, physical examination, or laboratory findings show that the disorder is the result of other physical illnesses, such as substance intoxication or withdrawal, toxin exposure, or multiple factors.

POD usually be diagnosed in the general ward or ICU, sometimes in emergency room, which should be distinguished from emergency agitation. Emergency agitation was defined as a Richmond Agitation–Sedation Scale (RASS) score of + 3 or + 4 or the administration of haloperidol during the PACU stay (Sessler et al. 2002).

### Missing values

Data cleaning was performed before the statistical analyses, and variables with more than 10% missing values were not included. For continuous variables with missing values (< 10%), the mean was used instead if the data were normally distributed, or the median was used if the data were nonnormally distributed. Patientes with missing categorical variable data were removed.

### Statistical analysis

Categorical variables are represented by the number of cases, and the chi-square test was used to compare 2 groups. Continuous variables are expressed as medians and interquartile ranges (IQRs), and the *t* tests or rank-sum tests were used to compare 2 groups. Stata MP 14.2 statistical software was used, and a *P* value < 0.05 was considered statistically significant. Univariate logistic regression was used to screen risk factors, and risk factors with a *P* value < 0.05 in the univariate logistic regression were included in the multivariate logistic regression analysis. Stepwise regression was also used to screen variables to construct the predictive models. All risk factors were dichotomized, and cutoff values for variables were based on commonly used clinical criteria or references.

The screened risk factors were introduced into R software version 4.1.2 (R Foundation for Statistical Computing), and the rms package was used to construct a nomogram for predicting the risk of POD in elderly patients undergoing orthopedic surgery. The internal validation of the model adopted the bootstrap method, and repeated sampling was performed 200 times for validation. The area under the receiver operating characteristic (ROC) curve (AUC) was used to evaluate the discrimination of the nomogram, a calibration curve was drawn to test the accuracy of the nomogram, and decision curve analysis (DCA) was used to evaluate the range of clinical validity of the nomogram.

## Results

From January 2019 to January 2022, 1015 elderly patients underwent orthopedic surgery, 4 of whom were excluded due to missing categorical variable data; a total of 1011 patients were included in the analysis.

### Clinical data between the POD and non-POD groups

In this study, 35 patients developed POD, for an incidence rate of 3.5%. The postoperative LOS of the POD group was significantly longer than that of the non-POD group (POD group: 13 days, non-POD group: 9 days). Age; sex; ASA classification; NYHA classification; preoperative cognitive impairment; pulmonary disease and arrhythmia status; preoperative Cr level, Hb level, and NLR; intraoperative blood loss; and blood transfusion status were significantly different between the 2 groups (Table 1).

**Table 1:** General characteristics of the patients with or without POD

Variable	POD (*n* = 35)	Non-POD (*n* = 976)	*P* value
Age (year)	83 (74, 86)	71 (67, 77)	0.000
Gender (male/female)	21/14	406/570	0.030
ASA (I/II/III/IV)	0/14/20/1	11/772/191/2	0.000
NYHA (I/II/III/IV)	10/19/6/0	314/626/36/0	0.000
Operative type (trunk bone fracture/limb bone fracture/joint replacement/tendon surgery/trunk and limbs)	3/22/10/0/0	186/610/177/1/2	0.395
Education (illiteracy/primary school/junior high school/senior high school and above)	17/15/3/0	556/332/72/16	0.609
Smoking (yes/no)	7/28	121/855	0.184
Alcohol (yes/no)	4/31	86/890	0.593
Comorbidity			
CAD (yes/no)	5/30	74/902	0.147
Hypertension (yes/no)	16/19	372/604	0.364
Arrhythmia (yes/no)	14/21	166/810	0.000
DM (yes/no)	1/34	90/886	0.196
Pulmonary disease	16/19	138/838	0.000
Precognitive impairment	9/26	10/966	0.000
Preoperative blood test			
Cr (mmol/L)	71 (61, 78)	59 (50, 69)	0.001
Hb (g/L)	104 (97, 118)	120 (107, 132)	0.000
WBC (*10^9^)	6.44 (4.13, 8.86)	6.44 (5.22, 8.11)	0.415
NLR (%)	6.34 (3.80, 9.63)	3.75 (2.50, 6.00)	0.000
Preoperative sedation (yes/no)	9/26	254/722	0.967
Anesthesia method (GA/SEA/NB/GA combined with LA /LA or basic anesthesia)	8/16/0/2/9/0	292/352/123/55/133	0.072
Bleeding (ml)	100 (20, 200)	200 (100, 300)	0.000
Fluid (ml)	1200 (600, 1700)	750 (500, 1200)	0.015
Surgical time (min)	115 (73, 158)	100 (70, 135)	0.274
Transfusion (yes/no)	15/20	178/790	0.000
Postoperative analgesia (no analgesia/systemic medication/NB/LA/systemic medication combined with others)	6/29/0/0/0	331/623/3/9/10	0.237
Postoperative_pain (yes/no)	5/30	167/809	0.662
ICU (yes/no)	2/33	29/947	0.355
LOS (d)	13 (11, 15)	9 (6, 14)	0.000

Data are median [lower quartile to upper quartile] and no. of cases

*POD* Postoperative delirium, *ASA* American society of Anesthesiologists, *NYHA* New York Heart Association, *CAD* Cardiac artery disease, *DM* Diabetes mellitus, *Cr* Creatinine, *Hb* Hemoglobin, *WBC* White blood cell, *NLR* Neutrophil-to-lymphocyte ratio, *GA* General anesthesia, *SEA* Spinal or epidural anesthesia, *NB* Nerve block, *LA* Local anesthesia, *ICU* Intensive care unit, *LOS* Length of hospital stay

### Univariate and multivariate logistic analyses

The univariate logistic regression results indicated that the factors correlated with POD were age, sex, ASA classification, NYHA classification, arrhythmia, preoperative pulmonary infection status, preoperative cognitive impairment status, Cr level, Hb level, WBC count, NLR, intraoperative blood transfusion status, and excessive infusion volume. Multivariate logistic regression revealed that age ≥ 75 years (odds ratio (OR) = 2.889; 95% confidence interval (CI), 1.149–7.264; *P* = 0.024), male sex (OR = 2.368; 95% CI, 1.066–5.261; *P* = 0.034),and preoperative cognitive impairment (OR = 13.587; 95% CI, 4.360–42.338; *P* = 0.000) were independent risk factors for POD (Table 2).

**Table 2:** Univariate and multivariate logistic analyses

Variable	Univariate logistic analysis			Multivariate logistic analysis		
	OR	95%CI	*P* value	OR	95%CI	*P* value
Age (years) (≥ 75 vs < 75)	5.707	2.644, 12.321	0.000	2.889	1.149,7.264	0.024
Gender (male vs female)	2.106	1.058, 4.191	0.034	2.368	1.066,5.261	0.034
ASA (III/IV vs I/II)	6.085	3.039, 12.186	0.000	1.960	0.818,4.698	0.131
NYHA (III/IV vs I/II)	5.402	2.110, 13.829	0.000	0.981	0.279,3.451	0.976
Operative type (hip replacement vs others)	1.458	0.952, 2.234	0.083			
Education (Illiteracy vs primary school and higher)	1.130	0.714, 1.788	0.601			
Alcohol (yes vs no)	1.335	0.461, 3.872	0.594			
Comorbidity						
CAD (yes vs no)	2.0332	0.766, 5.392	0.155			
Hypertension (yes vs no)	1.367	0.694, 2.692	0.365			
Arrhythmia (yes vs no)	3.253	1.621, 6.528	0.001	1.143	0.480,2.721	0.763
Pulmonary disease (yes vs no)	5.114	2.568, 10.185	0.000	2.132	0.945,4.806	0.068
DM (yes vs no)	0.290	0.039, 2.140	0.225			
Preoperative cognitive impairment (yes vs no)	33.438	12.535, 89.200	0.000	13.587	4.360,42.338	0.000
Preoperative blood test						
Cr (≥ 96 mmol/l vs < 96 mmol/l)	3.448	1.278, 9.307	0.015	1.999	0.623,6.411	0.244
Hb (< 110 g/L vs ≥ 110 g/L)	3.217	1.624, 6.372	0.001	1.640	0.684,3.933	0.268
WBC (≥ 10*10^9^ vs < 10*10^9^)	1.773	0.719, 4.372	0.214			
NLR (≥ 5 vs < 5)	2.886	1.372, 6.074	0.005	1.446	0.632,3.311	0.383
Anesthesia method (GA vs others)	0.725	0.344, 1.527	0.398			
Preoperative sedation (yes vs no)	0.984	0.455, 2.128	0.967			
Operation time (≥ 150 min vs < 150 min)	1.335	0.616, 2.893	0.465			
Bleeding (≥ 200 ml vs < 200 ml)	2.203	0.751, 6.467	0.151			
Fluid infusion (≥ 1200 ml vs < 1200 ml)	2.158	1.055, 4.4153	0.035	2.032	0.860,4.804	0.106
Transfusion (yes vs no)	3.362	1.688, 6.696	0.001	1.048	0.423,2.600	0.919
Postoperative pain (yes vs no)	0.807	0.309, 2.111	0.663			
Postoperative analgesia (systematic admission vs others)	1.303	0.809, 2.100	0.275			
ICU (yes vs no)	1.979	0.453, 8.645	0.364			

*OR* Odds rate, *CI* Confidence interval, *ASA* American society of Anesthesiologists, *NYHA* New York Heart Association, *CAD* cardiac artery disease, *DM* diabetes mellitus, *Cr* Creatinine, *Hb* Hemoglobin, *WBC* white blood cell, *GA* General anesthesia, *ICU* Intensive care unit

### Development of a predictive model for POD

**Fig. 1:**
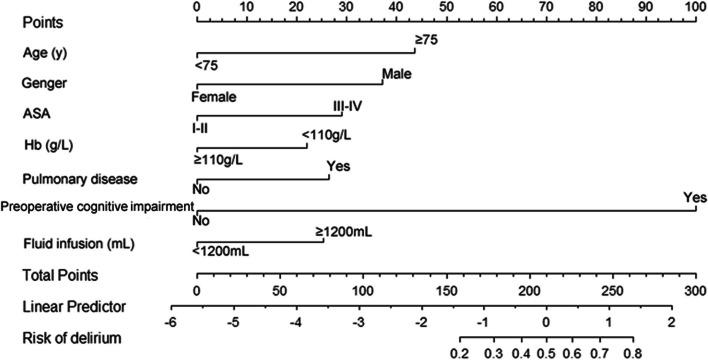
Nomogram for predicting the risk of POD in elderly patients undergoing orthopedic surgery

Multivariate logistic regression, combined with stepwise regression based on the least Akaike information criterion (AIC), indicated that 7 risk factors, i.e., age, sex, preoperative Hb level, ASA classification, preoperative pulmonary disease status, preoperative cognitive impairment, and intraoperative infusion volume, were used to construct the predictive model, and a score was assigned to each factor (Fig. 1). According to the nomogram, the higher the score is, the greater the risk.

### Internal validation of the prediction model

**Fig. 2:**
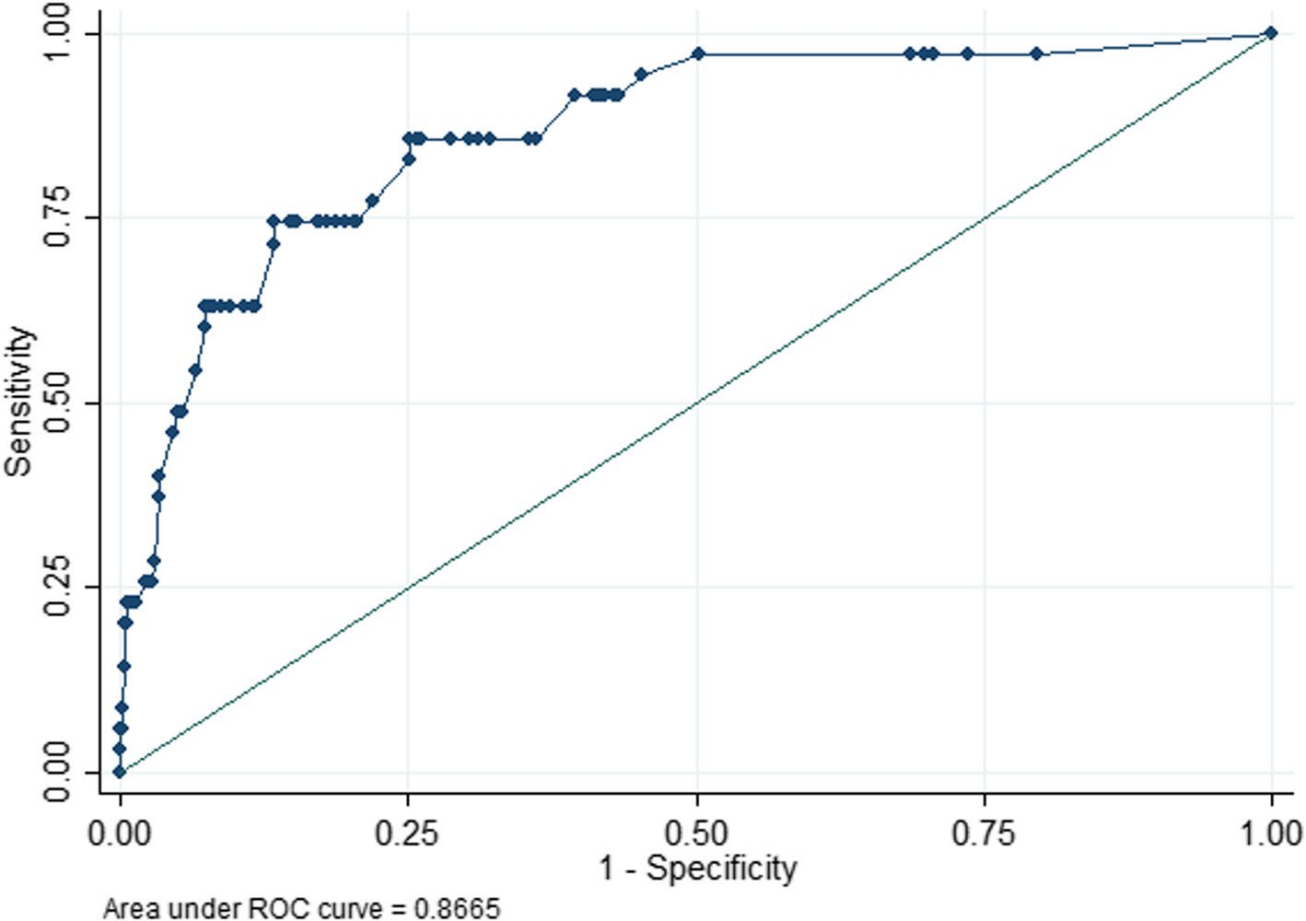
AUC of a nomogram for predicting POD in elderly patients undergoing orthopedic surgery. AUC, area under receiver-operating characteristic curve

**Fig. 3:**
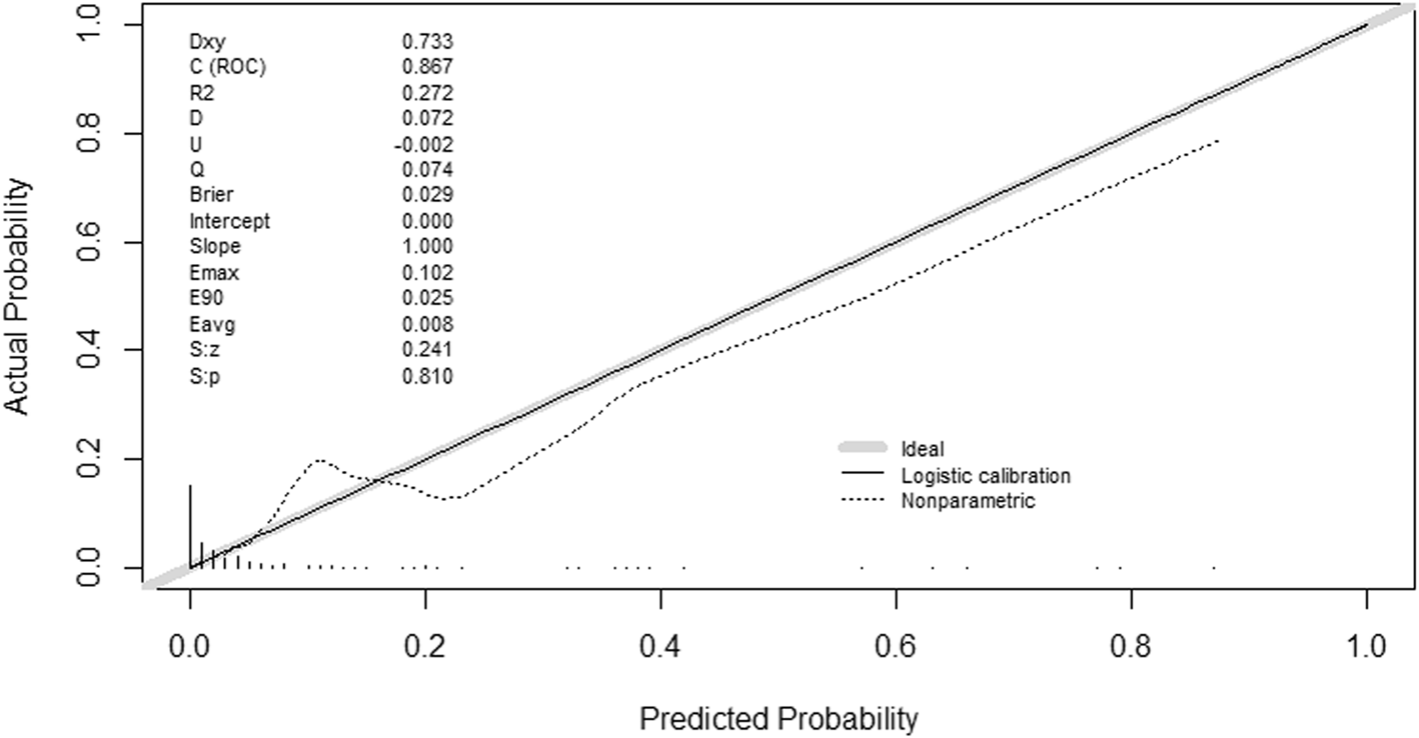
Calibration curve of a nomogram for predicting POD in elderly patients undergoing orthopedic surgery

**Fig. 4:**
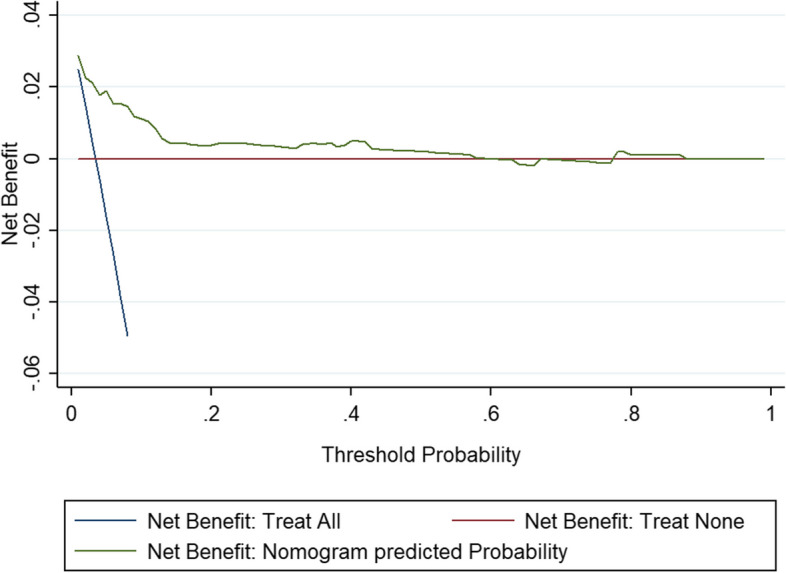
Decision curve analysis of nomogram for predicting POD in elderly patients undergoing orthopedic surgery

The AUC showed that the model had good discrimination (0.867) (Fig. 2), the calibration curve showed that the degree of fit was good (slope: 1.000, Brier: 0.029) (Fig. 3), and the DCA results showed that the nomogram model has an optimal net benefit ranging from 0.01 to 0.58, indicating good clinical applicability (Fig. 4). After using the bootstrap method (repeated sampling of 200 times), the internal validation results showed that the corrected C index was 0.846 (95% CI 0.839–0.853), which showed that there was no overfitting of this model, and the model was reproducible (Additional file 1).

## Discussion

POD is one of the most common complications after elective surgery in elderly patients. It prolongs the postoperative recovery time and LOS, affects postoperative quality of life, and increases morbidity and mortality risks (Urban et al. 2020; Lee et al. 2013; Susano et al. 2019; Brown et al. 2016). Older patients are more prone to complications after surgery due to aging, comorbidities, and frailty. Therefore, it is highly important to screen for risk factors for POD in elderly orthopedic surgical patients, carry out risk stratification, identify high-risk individuals early, and take corresponding preventive measures to reduce the occurrence of POD.

This study included data from 1011 patients, and the incidence of POD was 3.5%, which was relatively low compared with that in previous reports, possibly due to inconsistent diagnostic criteria or missed diagnoses. In this study, a nomogram was constructed that included age, sex, preoperative Hb level, ASA classification, preoperative pulmonary disease status, preoperative cognitive impairment status, and intraoperative fluid infusion volume; this nomogram showed good discrimination ability, accuracy, and clinical applicability in predicting POD in elderly patients who underwent orthopedic surgery.

This study showed that sex was an independent risk factor for POD, which is consistent with the results reported by Wu et al. (2021). Liang et al. also included male patients in a nomogram (Liang et al. 2015). In this study, male sex was an independent risk factor, possibly because the male patients in this study had more comorbid lung disease and cognitive impairment; however, the influence of sex on POD is currently controversial (Morrison et al. 2003). Many studies have shown that advanced age and ASA classification are high-risk factors for POD in elderly patients; elderly patients have more comorbidities, their functional reserve is lower, and their ASA classification is usually greater than grade III, and therefore, their tolerance to anesthesia and surgery is reduced, leading to an increased risk of POD (Liang et al. 2015; Yang et al. 2017; Kim et al. 2020; Zhang et al. 2019; Ahmed et al. 2014), which is consistent with the findings of this study. In this study, age ≥ 75 years and ASA grade ≥ III were two very important risk factors for POD, with scores of 43.6 and 29.1, respectively, in the nomogram, and preoperative cognitive impairment, which had a weight of 100 points in the nomogram, was the highest risk factor among all risk factors, which is consistent with the results of many studies (Kim et al. 2020; Zhang et al. 2019; Ristescu et al. 2021; Knaak et al. 2020; Silbert et al. 2015). Patients with preoperative cognitive impairment have neurological damage and decline, and the production of neuroinflammatory factors could increase during surgery; therefore, such patients are more prone to POD (Knaak et al. 2020). Preoperative anemia and pulmonary disease were risk factors for POD in this study. Anemia can lead to an insufficient blood supply and oxygen supply to the body, and lung disease affects the body’s intake of oxygen, resulting in reduced cerebral oxygen delivery and inadequate cerebral perfusion, which predisposes individuals to POD (Ali et al. 2021; Onuma et al. 2020; Clemmesen et al. 2019; Yang et al. 2020; Lima et al. 2021). Therefore, we should actively correct anemia and improve lung function before surgery.

Intraoperative fluid infusion volume can reflect the complexity of a surgery, and an increase in infusion volume indicates an increase in difficulty. An appropriate volume of fluid can maintain stable hemodynamics and ensure blood and oxygen supply to the brain. Fluid overload can also increase the incidence of POD. Therefore, intraoperative goal-directed infusion is recommended to reduce the incidence of postoperative complications (Wang et al. 2021).

In this study, the risk of POD was calculated based on a score generated using the prediction nomogram model (Fig. 1). For a male patient (score 37.2) aged over 75 years (score 43.6), an ASA III (score 29.1), COPD (score 26.5), cognitive impairment (score 100), and anaemia (Hb < 110) (score 22), the nomogram score was 258.4; if the intraoperative infusion was > 1200 ml (score 25.2), then the total nomogram score was 283.6, and the corresponding risk of POD was 88%, i.e., this elderly orthopedic surgical patient was highly likely to develop POD.

A nomogram can quantify and visualize logistic regression results, assign specific quantitative values, and provide individualized risk predictions for clinical adverse events (Eastham et al. 2002). The POD prediction model in this study included patient, surgical, and anesthesia factors and is applicable to all elderly patients undergoing orthopedic surgery, potentially helping medical staff detect such high-risk individuals earlier. However, this was a retrospective study conducted in 1 center, and there may be selection bias and regional and racial differences. Most POD cases are the low activity type, have atypical clinical symptoms, and are often overlooked by medical staff. Additionally, POD patients are mostly diagnosed through consultations with anesthesiologists or psychiatrists due to the poor diagnostic ability of orthopedic physicians in our hospital. Therefore, the results of this study support the development of nomograms for patients with severe POD. Moreover, the variables used for this study were initially measured during hospitalization, and no variables were measured 30 days or more after surgery. Therefore, subsequent multicenter studies with longer observation times and larger sample sizes are needed for validation.

## Conclusion

This study revealed 3 independent risk factors, i.e., advanced age, male sex, and preoperative cognitive impairment, and included an additional 4 risk factors (preoperative anemia, combined pulmonary disease, ASA grade, and intraoperative infusion) in a nomogram to predict the risk of POD after orthopedic surgery in elderly individuals. Internal validation of the nomogram by the area under the curve (AUC), calibration curve, and DCA showed that it can be used as a simple and effective tool for predicting POD.

### Supplementary Information

**Supplementary Material 1: Table S1.** Internal calibration.

## Data Availability

All data and materials are available from the corresponding author on reasonable request.
