# Supplementary material for: Nomogram for predicting the risk of postoperative delirium in elderly patients undergoing orthopedic surgery
Source: Perioper Med (Lond). 2024 May 4;13:34. doi: 10.1186/s13741-024-00393-9 (PMC11069318; doi:10.1186/s13741-024-00393-9)
Supplement: Supplementary file 1 — Supplementary Material 1: Table S1. Internal calibration. [file 13741_2024_393_MOESM1_ESM.docx]

Supplemental table 1 Internal calibration

| Item | Index.orig | Training | Test | Optimism | Index.corrected | n |
| --- | --- | --- | --- | --- | --- | --- |
| Dxy | 0.740544496 | 0.742612202 | 0.698306898 | 0.044305304 | 0.696239192 | 165 |
| R2 | 0.278173085 | 0.297828859 | 0.251277652 | 0.046551207 | 0.231621879 | 165 |
| Intercept | 0 | 0 | -0.259976755 | 0.259976755 | -0.259976755 | 165 |
| Slope | 1 | 1 | 0.897486859 | 0.102513141 | 0.897486859 | 165 |
| Emax | 0 | 0 | 0.07744259 | 0.07744259 | 0.07744259 | 165 |
| D | 0.074038863 | 0.079922691 | 0.06654469 | 0.013378001 | 0.060660863 | 165 |
| U | -0.001978239 | -0.001978239 | 0.001210816 | -0.003189055 | 0.001210816 | 165 |
| Q | 0.076017103 | 0.081900931 | 0.065333875 | 0.016567056 | 0.059450047 | 165 |
| B | 0.028483463 | 0.027978929 | 0.029397713 | -0.001418784 | 0.029902247 | 165 |
| g | 1.308639277 | 1.41157744 | 1.253899117 | 0.157678323 | 1.150960954 | 165 |
| gp | 0.046114041 | 0.047896054 | 0.044379025 | 0.003517029 | 0.042597013 | 165 |
